# Editorial: Emerging trends in pediatric pain

**DOI:** 10.3389/fpain.2023.1346075

**Published:** 2023-12-21

**Authors:** Kenneth Craig

**Affiliations:** Department of Psychology, University of British Columbia, Vancouver, BC, Canada

**Keywords:** pain, children, selfreport, nonverbal, facial

**Editorial on the Research Topic** Emerging trends in pediatric pain

Only recently have clinicians, investigators, and the public come to recognize unique requirements for assessment and treatment of pain in infants, young children, and adolescents as well as related longstanding inadequacies in clinical practice. Transformations in the experience, expression, and response to care through progression from infancy to adolescence (and continuing stages of life) require attention when providing treatment. One cannot talk to infants to either discover the nature of their painful distress or to provide solace, necessitating attention to nonverbal expression, and, while verbal capacities in older children and adolescents permit at least limited access to experience, careful attention to nonverbal expression in these populations allows a broader understanding of sensations, feelings, and thoughts. This series of six papers illustrates the necessity of incorporating consideration of the developmental stage of the person in an effort to understand and ameliorate painful distress. Cuviello et al., in their paper, *Regional blocks for pain control at the end of life in pediatric oncology* demonstrate the importance of attending to personal distress at the end of life at all stages of childhood. The paper by Bueno et al., *The effectiveness of repeated sucrose for procedural pain in neonates in a longitudinal observational study* demonstrates that standardized detailed behavioural observation reveals the benefits of sucrose administration in newborns during skin-breaking procedures. Similarly, but in older children, Bhatnagar et al., show the value of behavioural observation in their paper, *Assessing changes in range of motion in adolescent patients undergoing myoActivation*® *for chronic pain related to myofascial dysfunction.* Vigouroux et al., demonstrate the potential adverse effects of preconceptions concerning the pain of others in “*He told me my pain was in my head*”*: Mitigating testimonial injustice through peer support*. The risks that injustice appraisals will exacerbate emotional upheaval are evident in the paper by Daenen et al., *Youth baseline and state pain-related injustice appraisals are associated with emotional responses of anger and sadness: An experimental study.* The paper, *Development and expansion of a pediatric transitional pain service to prevent complex chronic pain*, by Isaac et al., demonstrates the benefits of systematically structuring services to ensure acute pain does not transition into a prolonged problem.

